# A New Antipruritic Remedy

**Published:** 1875-10

**Authors:** L. Duncan Bulkley

**Affiliations:** New York


### A NEW ANTIPRURITIC REMEDY.

BY L. DUNCAN BULKLEY, A. M., M. D., OF NEW YORK.

Eighteen months ago we presented to the profession the "*liquor picis alkalinus*,"* which had proved itself valuable in certain cutaneous affections in relieving itching in many instances, and whose value in this and other directions is daily becoming more evident. The failure of this as an antipruritic in some cases, as might be expected, has led us to the adoption of the compound here presented, which has thus far rendered inestimable service, but which has not, to my knowledge, been heretofore commonly known.

More than a year ago I noticed in a medical journal the item that equal parts of hydrate of chloral and gum camphor when rubbed well together are converted into a fluid substance—which observation was repeated in the *Boston Medical and Surgical Journal* of February 19, 1874, with the variation that the substances were to be mixed together, and the conversion would take place in a few days; no therapeutical suggestion was made in either instance. Stepping into a drug store, I immediately tested the truth of this, and found, after a few minutes' trituration in a mortar of powdered

---

* R.—Picis liquidæ, fl.drm. ij.; potass. causticæ. drm. j.; aquæ destill. fl. drm. v.; ft. liq. picis. alk.; use diluted. Archives of Scientific and Practical Medicine (Brown-Séquard,) February, 1873.

camphor and Schering's chloral, that in place of two crystalline substances a transparent colorless fluid existed, of the consistency of glycerin—no solvent having been added. It at once occurred to me that this would be of value in allaying itching, inasmuch as camphor acts ofttimes very gratefully when locally applied for this purpose, while I have long employed the chloral internally to the same end.

I, therefore, had some ointment prepared after the formula:

℞.—Pulv. gummi camph.,

Chloral hydrat., . . . . . . aa ʒj.

Ung. aquæ rosæ, . . . . . . . ℥j.—M.

Rub the chloral and camphor carefully together till fluid results; then add slowly to the ointment, mixing well.

This, when applied to the healthy skin, produces no effeet, but possesses great power in arresting itching without overstimulating the parts. It does not answer when the skin is at all broken; it is then neeessary to employ other less irritating agents, but the burning sensation caused on its first application lasts but a few moments, while the relief occasioned I have known to last for hours, or even a whole day.

This compound is soluble also in almond oil, alcohol, and ether to a considerable strength, as also in collodion. I have never employed it other than in the formula above given, except that I have ordered it of a less strength, half a drachm of each to the ounce, also increased to a drachm and a half of each to the same quantity. I should expect a solution of moderate strength in collodion would prove useful in localized itching. I will give two or three illustrative cases:

Mrs. J. C. L., aged 34, seven months pregnant, was distressed with a most intense itching which commenced six weeks previous to her being referred to me by Dr. H. B. Sands, on December 4, 1873. The itching was first noticed on the left arm near the shoulder, then on the right and on both shoulders behind, and soon the outer surfaces of the legs and the region of the waist became affected. When first seen, her condition was indeed pitiable, the more so as she had become convinced that the distress would last until confinement. Her general condition was poor; she looked worn and nervous; pulse 104, and weak; tongue coated; appetite very poor, and bowels irregular in action. She gave the history of being subject to neuralgia, and of having had a similar attack of pruritus when not pregnant nine months previous, which disappeared on the use of six sulphur baths. The skin when seen presented only the appearances due to scratching, namely, excoriated points and lines, resembling those seen in phthiriasis, but no traces of pediculi could be found nor indeed could any suspicion of this exist.

Diuretic and tonic treatment seemed to effect very little, but some relief was afforded by the alkaline tar-wash before referred to, diluted about one part to sixteen of water. This was only temporary, and chloral and bromide of potassium internally failed to procure even a quiet night. The chloral and camphor in ointment, a drachm each to the one ounce, was then prescribed, and the relief obtained was immediate and permanent; that is, with the application repeated every few hours at first, for she never really suffered from the itching after its first use, and in six days she had no further need of it. It is but right to state that she continued the same internal treatment as before, but she did not require any medicine to induce sleep. On the disappearance of the cutaneous symptoms a hoarseness occurred, evidently nervous in origin, which lasted until delivery, as it had accompanied also a previous pregnancy to the end.

Mrs. F. C. M., aged 32; a lady of highly nervous organization who was in the habit of greatly overworking in a literary line; has had several attacks during the last two and a half years of a very peculiar pruritus extending down the right arm and on the right shoulder behind to the median line. The itching and burning she describes as horrible, and the skin was found to be hard and scratched. On former occasions she had obtained very great relief from the liquor picis alkalinus diluted, with internal medication, but nervous exhaustion had been repeated so often by excessive literary work that all the old measures failed to give relief at the last attack I witnessed. The nerve-distress is further shown by a partial writer's cramp.

She was directed to abstain from using the right hand as much as possible, substituting the left, and was given the chloral and camphor ointment, a drachm each to the ounce. The relief following the first application was prompt and perfect, and a few days sufficed to remove the itching entirely, she using the same internal treatment which before proved ineffectual; no return of the pruritus has yet occurred after the expiration of several months. Whether on a recurrence of the skin trouble the remedy will still exercise as beneficial an influence, it is impossible to say, but the relief afforded in this instance was as striking as it was gratifying. I will give one more case:

Mrs. B———, aged 56, a large, fleshy lady, was kindly referred to me by Dr. A. L. Loomis, for a *pruritus vulvæ* which had lasted six months. During the previous summer she began to have more or less irritation about the vulva, and had scratched herself to relieve it, continuing to do so till seen by me in December, 1873. A very large number of remedies had been most judiciously applied, with but very little relief to the distressing itching, and on inspection the parts were found to be greatly thickened and discolored with more or less excoriation reaching down on the thighs and toward the anus. The clitoris was enlarged and elongated, but no follicular condition of the mucous membrane, no cervical erosions, nor any apparent cause of the trouble could be discovered. The urine was examined and gave no indications of glycosuria. The irritation and itching of the whole region she said were beyond description, and her troubled expression of face told the same story. She was then keeping the bed and using hot hip-baths night and morning with some comfort. These latter were directed to be exchanged for fomentations with very hot water frequently repeated, and iron, quinine, and Fowler's solution were given, also a diuretic mixture later.

Some considerable amelioration was obtained subsequently by the alkaline tar used in the other cases, but soon this seemed to have lost its effect, and various other applications failed to cause improvement. After about a week's treatment, the camphor and chloral ointment was prescribed with the happiest results, the itching disappearing entirely sometimes for seven or more hours after its application, while, in the meantime, the patient gained in physical condition, and the thickened state of the parts which had been caused and kept up by scratching subsided, and she was able to be up and around with a great degree of comfort. There still remains some itching at times, and the patient has just left for Europe for change of scene, etc., though for the last two months or so the irritation has been very slight. The relief afforded by the ointment in this case far exceeded that obtained from any other measure attempted.

In using this remedy, the only caution I know of is to see that the parts to which it is to be applied are not greatly, if at all, excoriated by scratching, as the application is then pretty painful, and the unprotected dermia is irritated and possibly inflamed thereby.

From its success in these three forms of itching here exemplified, namely, that of pregnancy, that of the vulva, and that due to a pure neurosis as in the second case, I would suggest the employment of the camphor and chloral compound in the general pruritus attending the senile alterations in the skin; also in anomalous cases and in those of chronic papular eczema or lichen, where itching is a prominent feature. Likewise in the *pruritus hiemalis* lately described by Dr. Duhring, of Philadelphia, in the *Philadelphia Medical Times*, of which I have seen some instances.

The ointment loses strength on standing exposed, and should be made fresh very frequently.

